# Towards a better understanding of the novel avian-origin H7N9 influenza A virus in China

**DOI:** 10.1038/srep02318

**Published:** 2013-07-30

**Authors:** Yongbo Wang, Zhangyan Dai, Han Cheng, Zexian Liu, Zhicheng Pan, Wankun Deng, Tianshun Gao, Xiaotong Li, Yuangen Yao, Jian Ren, Yu Xue

**Affiliations:** 1 Department of Biomedical Engineering, College of Life Science and Technology, Huazhong University of Science and Technology, Wuhan, Hubei 430074, China; 2 School of Chemistry and Molecular Biosciences, University of Queensland, Brisbane, Queensland 4027, Australia; 3 State Key Laboratory of Biocontrol, School of Life Sciences, Sun Yat-sen University, Guangzhou, Guangdong 510275, China; 4 These authors contributed equally to this work.

## Abstract

Recently, a highly dangerous bird flu has infected over 130 patients in China, and the outbreak was attributed to a novel avian-origin H7N9 virus. Here, we performed a systematic analysis of the virus. We clarified the controversial viewpoint on neuraminidase (NA) origin and confirmed it was reassorted from Korean wild birds with higher confidence, whereas common ancestors of pathogenic H7N9 genes existed only one or two years ago. Further analysis of NA sequences suggested that most variations are not drug resistant and current drugs are still effective for the therapy. We also identified a potentially optimal 9-mer epitope, which can be helpful for vaccine development. The interaction of hemagglutinin (HA) and human receptor analog was confirmed by structural modeling, while NA might influence cellular processes through a PDZ-binding motif. A simplified virus infection model was proposed. Taken together, our studies provide a better understanding of the newly reassorted H7N9 viruses.

On 29 March 2013, Chinese Center for Disease Control and Prevention (China CDC) isolated and confirmed a new influenza A (H7N9) virus that had infected three Chinese patients, with two from Shanghai and one from Anhui Province. It's the first time that H7N9 viruses infect humans^1^,^2^. The Chinese media (Ecns.cn) reported the considerably rapid spread of the virus-caused flu. Before April 12, all infected cases were detected only in or near to the Yangtze river delta, while one seven-year-old girl in Beijing and two residents in Henan Province were newly confirmed with H7N9 bird flu on April 14. In particular, the husband of a Shanghai woman who died as a result of H7N9 infection on April 3 was also confirmed to be infected with the bird flu virus on April 11, and the human-to-human transmission can still not be fully excluded. In this regard, the anti-viral therapy and vaccines are urgently needed, whereas more analyses will be greatly helpful for better understanding the H7N9 subtype viruses^3^,^4^,^5^,^6^,^7^. Till May 6, H7N9 viruses have infected up to 129 patients and killed 31 of them in China.

All influenza A viruses encode 8 genes, which can be translated into 11 distinct proteins by different open reading frames (ORFs)^8^. Also, influenza A viruses have been classified into distinct subtypes based on two surface glycoproteins of hemagglutinin (HA) and neuraminidase (NA)^9^. For six internal genes, the M gene generates two different matrix proteins (M1 and M2), while the nucleoprotein (NP) and RNA polymerases transcribed from PA (encodes PA protein), PB1 (encodes PB1 and PB1-F2) and PB2 (encodes PB2) form protein complexes that interact with viral RNAs^8^. In addition, two non-structural proteins (NS1 and NS2/NEP) are encoded by the NS gene^10^. It has been reported that influenza A viruses are responsible for both seasonal and periodic world-wide flu outbreaks^6^,^11^,^12^.

Accurate determination of the origin of highly pathogenic avian influenza (HPAI) H7N9 viruses is the most crucial problem for preventing pandemic^3^,^6^,^7^,^13^. It's still not exactly known where H7N9 virus originated, but several lines of evidences have supported its avian-origin. On April 11, researchers from China and Japan published their back-to-back results for early findings of H7N9 viruses, separately^3^,^6^. In Gao's study, H7N9 viruses were isolated and sequenced from three Chinese patient samples, while phylogenetic analyses supported a triple reassortant model that H7N9 might be reassorted by H7N3 HA in Zhejiang duck, H7N9 NA in Korean wild bird, and six internal genes (PB2, PB1, PA, NP, M and NS) of H9N2 in Beijing brambling^6^. Kageyama's results largely supported this model, whereas they drew a different conclusion that NA might be reassorted from mallard in Czech Republic^3^. Both analyses reported an R294K mutation of NA in A/Shanghai/1/2013 to be resistant to Oseltamivir (Tamiflu)^3^,^6^,^14^. In the NA stalk region of all H7N9 viruses, both studies detected a five amino acids deletion (69-73aa), which is associated with increased virulence^3^,^6^,^15^. In particular, both analyses revealed mutations in known receptor-binding sites (RBSs) of HA may increase the binding affinity of H7N9 viruses to human-type receptors^3^,^6^.

In this work, we first performed phylogenetic analyses for each gene in newly sequenced HPAI H7N9 viruses, respectively. The controversial viewpoint of Korea or Czech Republic-origin of NA gene was carefully evaluated and clarified. Also, we estimated the time of the most recent common ancestor (TMRCA) of each HPAI H7N9 gene, with a Bayesian Markov chain Monte Carlo (MCMC) approach^11^,^16^. The results suggested that common ancestors of the genes were originated in recent years ago, and the mutation rates of genes in HPAI H7N9 virues are greater than in swine-origin influenza A (H1N1) viruses (S-OIVs)^11^. Based on previous studies^3^,^6^ and our phylogenetic results, a more rigorous model of “three-step reassortant” was raised for the avian-origin H7N9 virus. By sequence alignment and analysis, genetic variations in HPAI H7N9 viruses were systematically characterized and can serve as a useful resource for further functional experiments. The structural modeling suggests that current anti-viral drugs such as Oseltamivir, Laninamivir, and Zanamivir are still effective for the therapy. Furthermore, combined with sequence-based prediction and structural docking approach, we identified a potentially optimal 9-mer epitope, which might be helpful for vaccine development. Moreover, we detected a N9-conserved C-terminal class 2 PDZ-binding motif in H7N9 NA protein, which may regulate cellular processes through interacting with PDZ domain proteins. In addition, we confirmed the interaction between HA and avian/human receptor analog by structural modeling. Finally, a potential model for HPAI H7N9 virus infection was raised. Taken together, our analyses are helpful for better understanding the new avian-origin H7N9 viruses, whereas the results can be useful for further experimental consideration.

## Results

### Phylogenetic analysis of avian-origin H7N9 viruses

**Figure 1:**
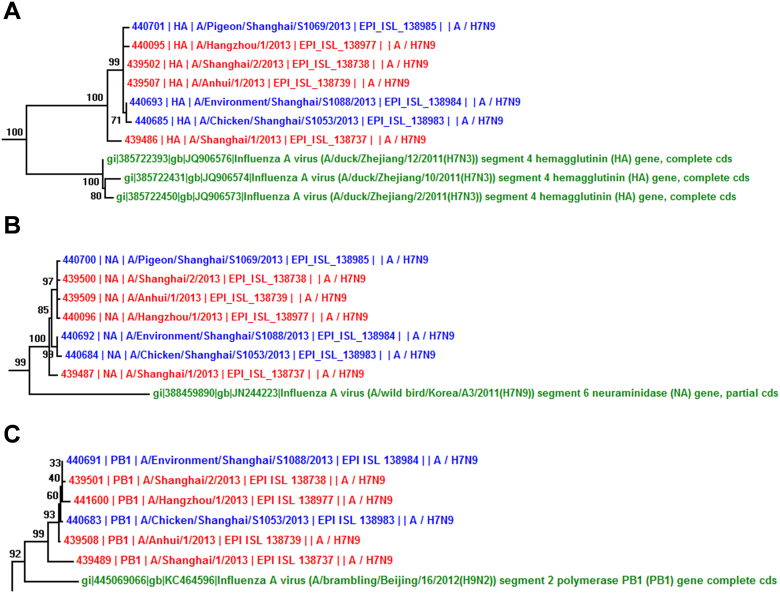
Phylogenetic analyses of H7N9 virus genes. The MP trees were present for (A) HA and (B) NA. (C) The NJ tree was visualized for PB1. The HPAI viruses in patients were marked in red, while HPAI viruses from non-human samples were marked in blue. The nearest genes from LPAI viruses were shown in green. More detailed phylogenetic trees were shown in Supplementary Fig. S1.

In this work, Neighbor-Joining (NJ) trees for eight genes of newly sequenced H7N9 viruses were constructed (Supplementary Fig. S1). To avoid any bias, maximum parsimony (MP) trees were also performed for HA and NA genes (Fig. 1a,b, Supplementary Fig. S1a,b). The NJ trees of HA and NA generate the same results (Supplementary Fig. S1c,d). Although different tree-constructing methods were used, our analyses are highly consistent with Gao's results^6^, which suggested that HA was reassorted from H7N3 in Zhejiang duck (Fig. 1a, Supplementary Fig. S1a) and NA was from H7N9 in Korean wild bird (Fig. 1b, Supplementary Fig. S1b). Also, our phylogenetic results of six internal HPAI genes do not fully support the Gao's hypothesis that all of them were originated from Beijing brambling^6^, because only three genes of PB1 (Fig. 1c, Supplementary Fig. S1e), PB2 (Supplementary Fig. S1f), and PA (Supplementary Fig. S1g) in HPAI H7N9 viruses are nearest from the H9N2 sample of A/brambling/Beijing/16/2012, and the results were confirmed in a recent analysis^16^. The other three genes of NP (Supplementary Fig. S1h), M (Supplementary Fig. S1i) and NS (Supplementary Fig. S1j) are not nearest from Beijing brambling, and might be originated from poultries in the Yangtze river delta.

As previously described^11^,^16^, a Bayesian MCMC method was used for estimating the TMRCA, the duration of unsampled diversity, and the evolutionary rate of each gene (Table 1). We observed that the common ancestors of HPAI H7N9 genes existed between May 2011 (NP) and July 2012 (PA), with a reassortant period of nearly 13 months (Table 1). The results are similar with a recent studies, which reported that the TMRCA of NA is the earliest and the TMRCA of PA is the latest in HPAI H7N9 viruses^16^. We confirmed the latest origin of PA, but further demonstrated that NP has the earliest origin (Table 1). In contrast, the TMRCAs of genes in S-OIVs ranges from March 2008 to September 2008, with only a duration of 6 months for reassortment^11^. In this regard, the reassortment of the HPAI H7N9 virus underwent a much longer period than the S-OIV. Also, by analyzing the duration of unsampled diversity, we observed that the common ancestor of HPAI H7N9 outbreak with the nearest low pathogenic avian influenza (LPAI) viruses existed from 0.63 to 3.27 years ago (Table 1), while the TMRCA of S-OIV with closest related virues ranges from 9.24 to 17.15 years ago^11^. Thus, in contrast with the poor surveillance of the S-OIV circulating, the epidemic surveillance for avian influenza viruses has been much improved. In addition, we revealed that the evolutionary rates of HPAI H7N9 virus genes (4.06 to 7.77 * 10^−3^ substitutions per site per year) are considerably greater than S-OIV (2.59 to 3.67 * 10^−3^ substitutions per site per year)^11^. In this regard, avian-origin H7N9 viruses evolve more rapidly and might be more dangerous to reassort HPAI viruses. Thus, a more rigorous surveillance will be helpful for further preventing the pandemic outbreak. In addition, the phylogenetic analysis is not helpful for calibrating a more precisely reassortant model, because the time scale is too short that the reassortment started around November 2012 (the data that A/brambling/Beijing/16/2012 was sequenced) and ended in February 2013.

### A landscape of genetic variations in H7N9-infected patient samples

**Figure 2:**
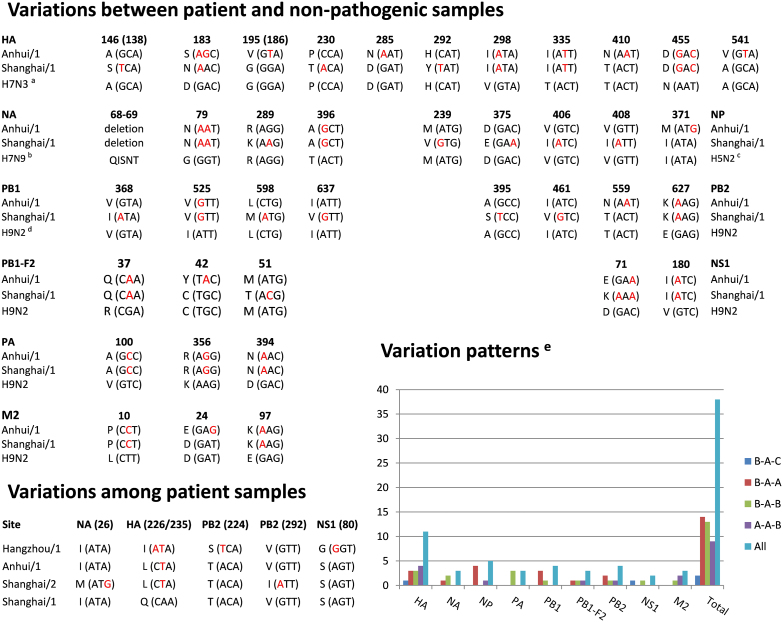
A variation map of HPAI H7N9 genes in patient samples. We analyzed variations between patients and non-pathogenic samples and among 4 patients, respectively. The variation patterns were also counted. (a) The benchmark HA sequence was taken from A/duck/Zhejiang/11/2011(H7N3); (b) Using identical positions of alignment results for 5 Korean wild bird H7N9 NA sequences as the background; (c) From A/chicken/Hebei/1102/2010(H5N2); (d) From A/chicken/Zhejiang/607/2011 (H9N2); (e) The statistics of variation patterns confirmed that H7N9 virus in A/Shanghai/1/2013 evolves more rapidly.

A number of genetic mutations or variations have been detected in recent studies^3^,^6^. Gao et al. observed a receptor specificity changing variation of Q226L in HA, an anti-viral resistance variation of R294K in NA, and a ferret-transmissible variation of I368V in PB1 among three patient samples^6^. All three samples have a 5aa deletion in the NA stalk region^6^, and this result was also confirmed by Kageyama's analysis^3^. Because the time was limited and only three strains were analyzed, mutations or variations might not be fully demonstrated. Here, we performed a more comprehensive analysis of non-synonymous variations between patient and non-pathogenic samples (Fig. 2). We defined variation patterns among the A/Shanghai/1/2013 group, non-pathogenic group, and A/Anhui/1/2013 group as 4 types, including B-A-C (one variation in the A/Shanghai/1/2013 group and another variation in the A/Anhui/1/2013 group), B-A-A, B-A-B, and A-A-B. In addition, we aligned sequences of 4 patient samples together for each protein separately to identify non-synonymous variations among patient samples. Totally, we characterized that point non-synonymous variations occur at up to 38 positions of 9 viral proteins, while M1 and NS2 are not mutated (Fig. 2). The 5aa deletion in NA was also detected. The variation patterns were analyzed, while the B-A-A type variations are predominant. Thus, the variation rate of A/Shanghai/1/2013 is greater than other patient samples^6^ (Fig. 2). From multiple alignment results of 5 NA sequences in Korean wild bird samples, non-synonymous variations occur at 66 positions and implicate a considerably high evolutionary speed. However, analysis of multi-alignments of 4 patient samples only identified 3 non-synonymous variations. Thus, NA gene does not exhibit an accelerated evolution after infecting humans. Furthermore, we identified that non-synonymous variations occur at 5 additional positions among 4 patient samples (Fig. 2). All well-documented drug resistant, virulence increasing and viral protein function-changing variations were fully covered by our results^3^,^6^. Other uncharacterized variations can be useful for further experimental consideration.

### Structural modeling suggests most variations are not drug-resistant

**Figure 3:**
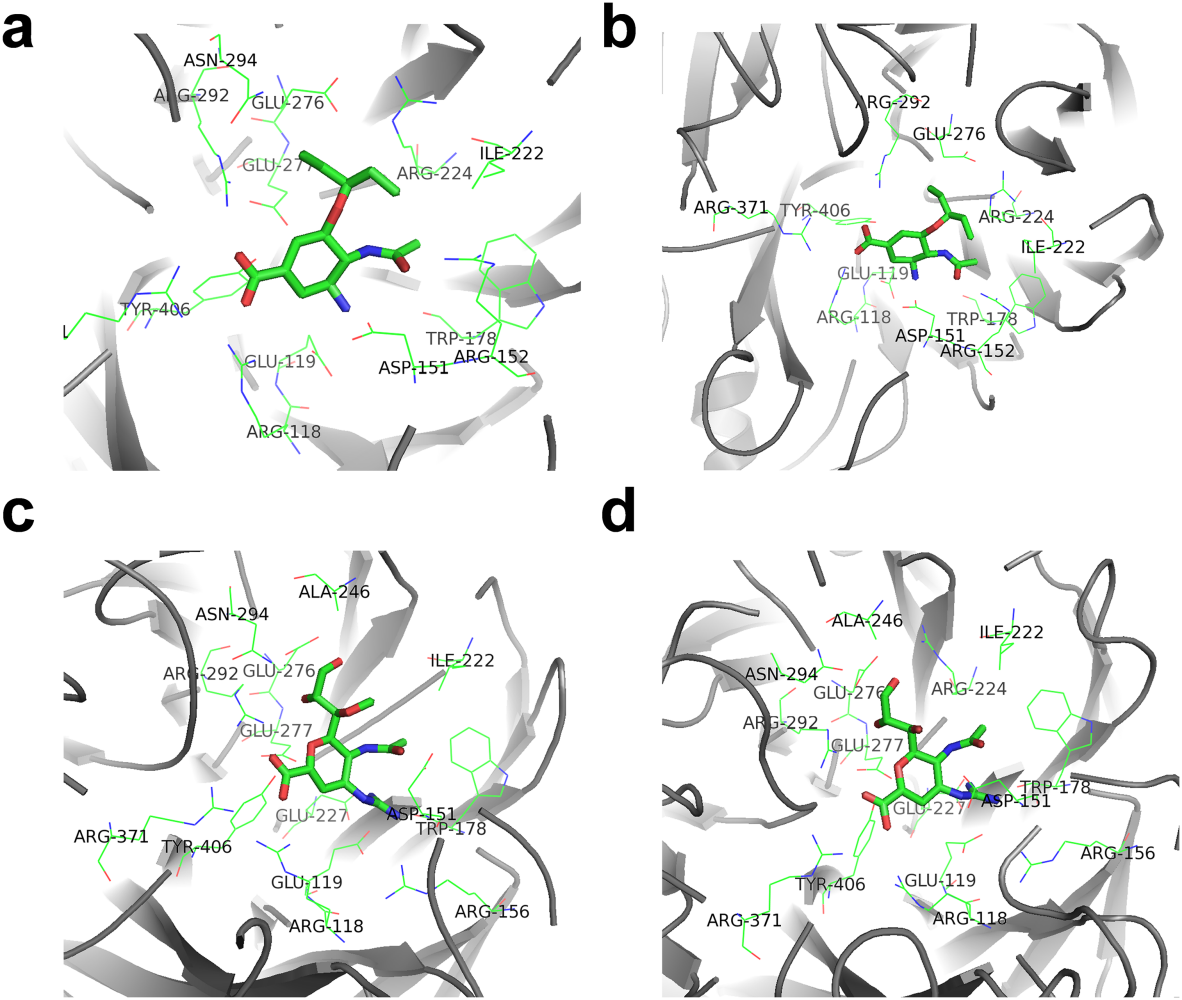
The structure modeling of NA-drug interactions. (a) The original NA-Oseltamivir complex from 2QWK^17^; Other complex structures are modeled for H7N9 subtype, including (b) NA-Oseltamivir, (c) NA-Laninamivir and (d) NA-Zanamivir. The drug-interacting residues (≤ 4 Å) were highlighted and labeled.

**Figure 4:**
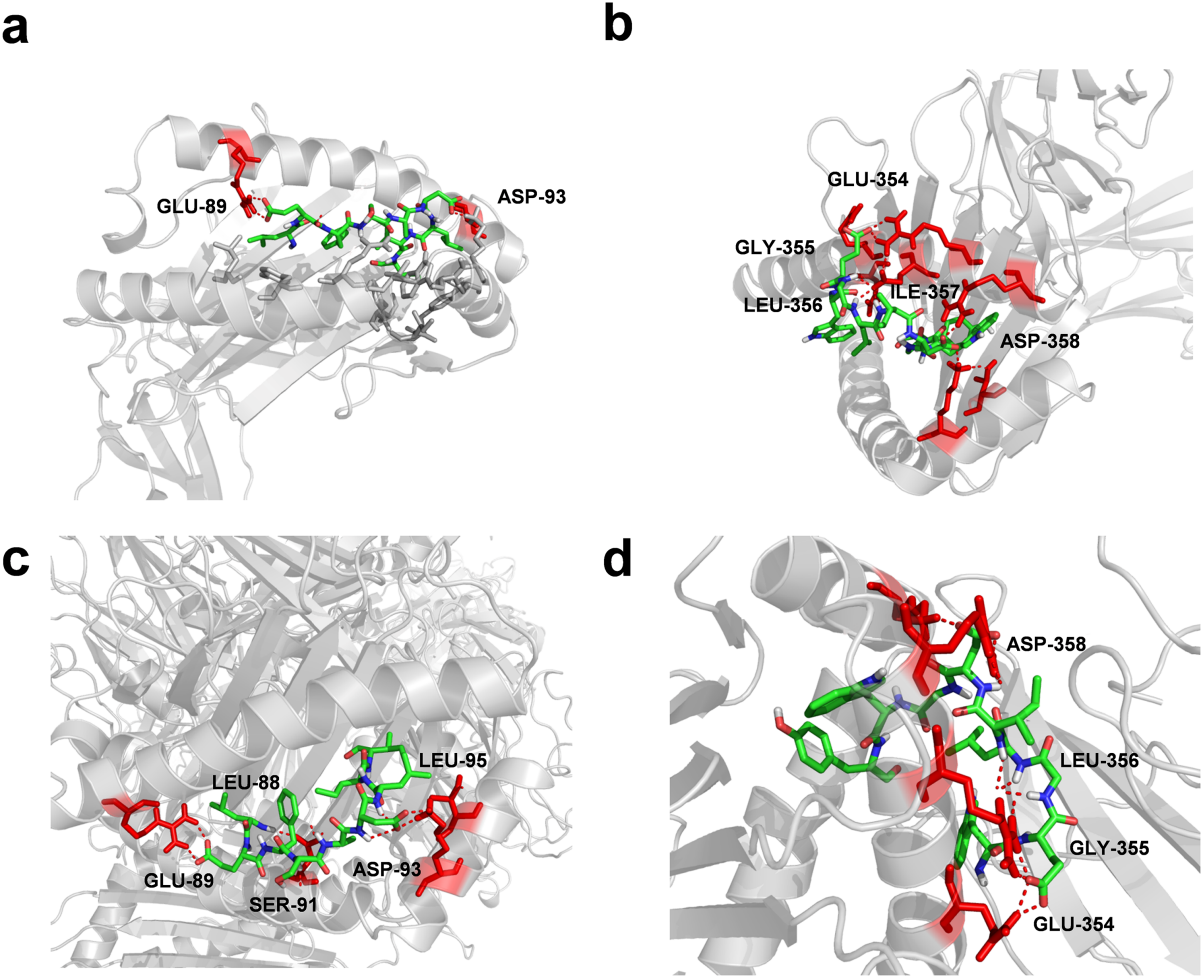
The molecule docking of HLA-B*4405, DM1-TCR and predicted epitopes. The key interacting residues in epitopes were labeled, while their binding sites in HLA-B*4405 or DM1-TCR were highlighted in red. (a) HLA-B*4405/epitope 88; (b) HLA-B*4405/epitope 353; (c) DM1-TCR/HLA-B*4405/epitope 88; (d) DM1-TCR/HLA-B*4405/epitope 353.

Anti-viral therapy with drugs might be the most effective treatment for H7N9 bird flu before vaccine development^3^,^4^,^5^,^6^,^7^. Because NA evolves rapidly at least in wild birds, it will be very dangerous if anti-viral resistance is conferred by genetic variations. Indeed, an R294K variation of NA in A/Shanghai/1/2013 is known to influence the NA-Oseltamivir (Tamiflu) interaction through disrupting the internal salt link between E276 and R224, and generate anti-viral resistance^3^,^6^. To test whether there are more drug-resistant variations, a previously characterized 3D structure of N9 NA-Oseltamivir complex (2QWK, Fig. 3a)^17^ was used as a template for modeling H7N9 NA-Oseltamivir interaction (2QWK, Fig. 3b). Also, two structures of 2009 pandemic H1N1 NA-Laninamivir (3TI3), NA-Zanamivir were adopted (3TI5) for modeling the interactions of H7N9 NA and the two drugs, respectively (Fig. 4c,d)^18^. As previously described^18^, key residues for drug interaction were directly determined and visualized from the structures, with a distance of ≤ 4 Å to drugs (Fig. 3). Totally, we identified 16 drug-interacting residues that are present in at least one complex (Table 2). The comparison of NA-Oseltamivir and H7N9 NA-Oseltamivir complexes suggested that most interacting residues are not changed, and the distances to drug are slightly increased (Table 2). Thus, it can be expected that the binding affinity of H7N9 NA-Oseltamivir will be only moderately weaken. From the results, we did not observe any additional drug resistant variations, except the R294K in A/Shanghai/1/2013 (Table 2). Our results can be used as a reference for further detection of potential drug resistance if new variations appear.

### Prediction of potential peptide epitopes for further vaccine design

As an important surface protein, HA is the major molecule for vaccine developed^19^,^20^. In a recent study, Su et al. performed a structural docking analysis of H1N1 and H5N1 epitopes that bind with HLA-B*4405 and DM1-TCR/HLA-B*4405 complex^19^, because T cell-mediated responses can be greatly enhanced by DM1-TCR/HLA-B*4405/epitope complex^21^. Using a sequence-based tool^22^, we totally predicted H7N9 HA with 8 T cell epitopes that potentially interact with HLA class I B44 supertype molecules (Table 3). As previously described^19^, each predicted peptide was first docked to HLA-B*4405, and HLA-B*4405/peptide complex was then docked to DM1-TCR. The results were ranked based on calculated binding energy scores for docking to HLA-B*4405 (Table 3). Thus, although epitope 441 achieved the highest score from the sequence-based prediction, it's not an optimal epitope for its high binding energy (Table 3). From the docking results, we observed that both epitope 88 and 353 are embedded in the binding groove of HLA-B*4405 (Fig. 4a,b). However, epitope 88 does not directly interact with DM1-TCR (Fig. 4c), while epitope 353 interacts with both DM1-TCR and HLA-B*4405 via E354, G355, L356 and D358 (Fig. 4d). Thus, it can be expected the DM1-TCR binding will increase the binding energy for epitope 88. Indeed, the binding energy of HLA-B*4405/epitope 88 was significantly enhanced from −904.6 to −643.9 in DM1-TCR/HLA-B*4405/epitope 88, while the results for epitope 353 are slightly changed (Table 3). Taken together, the epitope 353 is an optimal candidate for further optimization or vaccine design.

### H7N9 virus may increase virulence through a PDZ-binding motif at NA C-terminus

**Figure 5:**
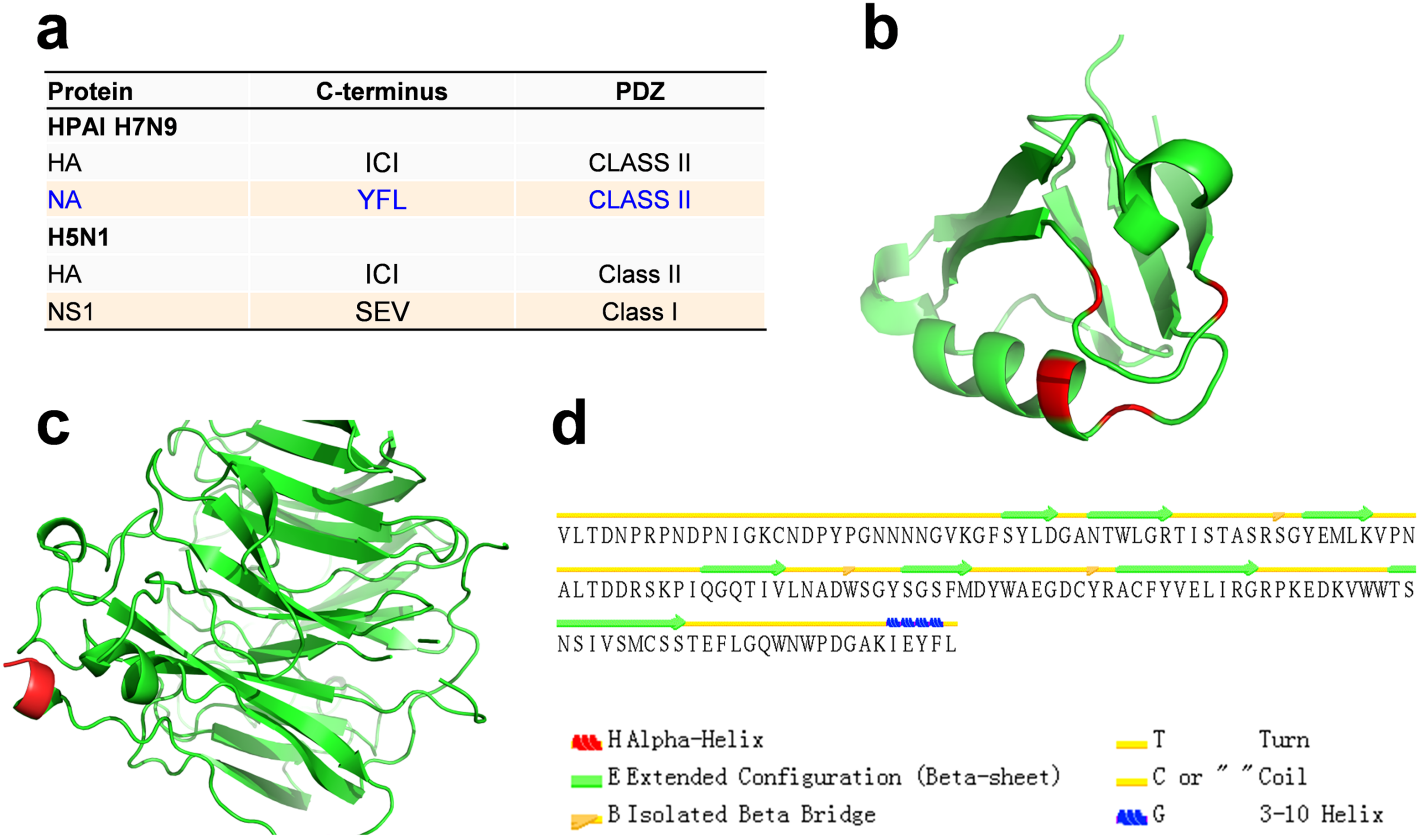
The analyses of PDZ-binding in influenza A virus. (a) The predicted PDZ-binding motifs in HPAI H7N9 and H5N1; (b) Interacting residues in NS1-binding PDZ domain (red); (c) The PDZ-binding motif in H7N9 NA (red); (d) Secondary structures of H7N9 NA C-terminus.

In Kageyama's results, a 218–230 deletion was detected at the H7N9 NS1 C-terminus^3^. This deletion removes a well characterized PDZ-binding motif, which determines the virulence by interacting with cellular PDZ domain proteins at least in H1N1 and H5N1, but does not influence the viral replication^10^,^23^,^24^. Thus, lack of the PDZ-binding motif will be expected to decrease the pathogenicity of H7N9 viruses^3^. However, this result can not interpret why the fatal rate is high in China. From ELM (Eukaryotic Linear Motif) resource, we obtained three known C-terminal PDZ-binding motifs, including S/T-X-A/C/V/I/L/F$ (class 1, $: the end of the sequence), V/L/I/F/Y-X-A/C/V/I/L/F$ (class 2), and D/E-X-A/C/V/I/L/F$ (class 3)^25^. We scanned all proteins in H5N1 and H7N9 viruses with the three motifs (Table 4). We observed that almost all HA proteins contain C-terminal class 2 PDZ-binding motifs (98.3% in H5N1 and 100% in H7N9) (Table 4, Fig. 5a), whereas 87.9% of H5N1 NS1 proteins have a class 1 motif (Table 4, Fig. 5a). Although 52.9% of H7N9 NS1 genes contain a class 2 binding motif, this motif only presents in LPAI H7N9 viruses (Table 4). Unexpectedly, most of H7N9 NA sequences (95.2%) have a class 2 motif (YFL) (Table 4, Fig. 5a), which is conserved in almost all N9 subtype NA proteins and may still regulate the virulence even if the NS1 does not interact with PDZ domain proteins any longer. The 3D structure of a typical NS1-binding PDZ domain in PDlim2 (3PDV) was visualized, while the binding residues were shown (Fig. 5b). Also, the PDZ-binding motif in H7N9 NA structure modeled from NA-Oseltamivir complex was highlighted (Fig. 5c). The secondary structure of H7N9 NA C-terminal was determined as a 3aa helix by a secondary structure assignment software of STRIDE (Fig. 5d)^26^.

## Discussion

**Figure 6:**
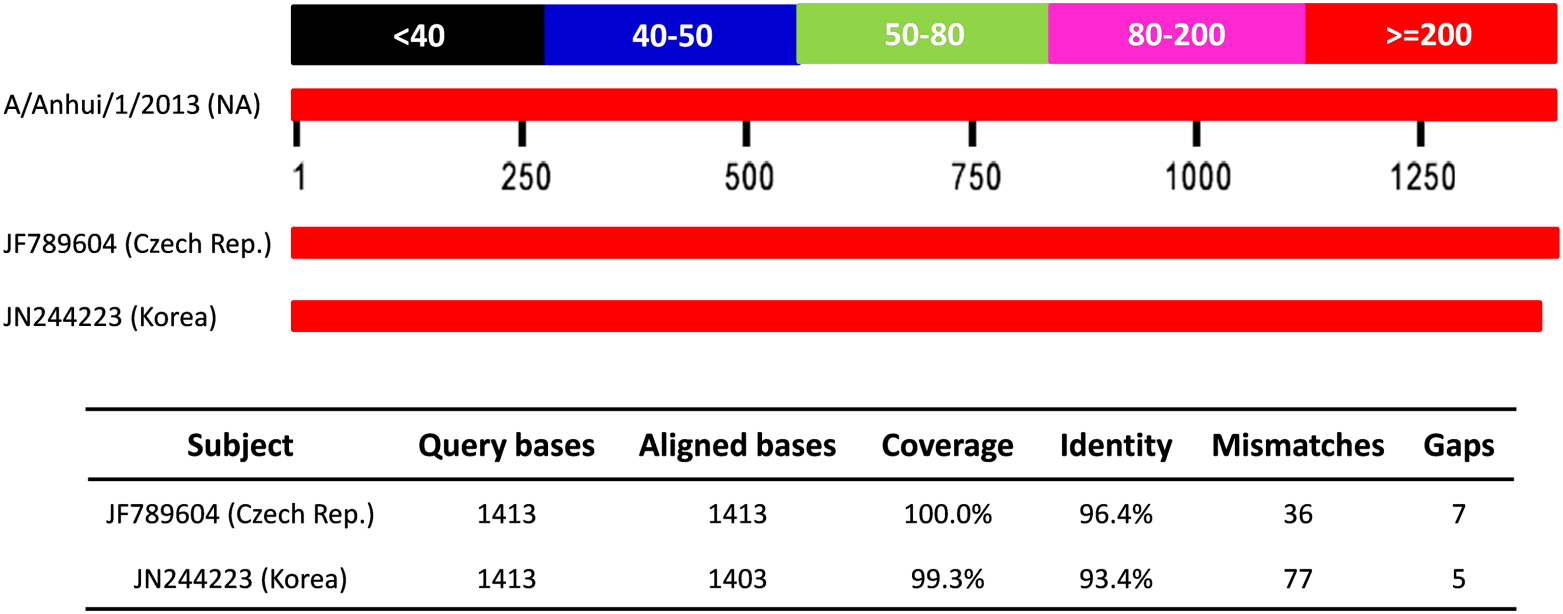
The sequence alignment of HPAI H7N9 NA with the sequences of A/mallard/Czech Republic/13438-29K/2010 (JF789604, H11N9) and A/wild bird/Korea/A3/2011 (JN244223, H7N9).

In this work, we first clarified a controversial viewpoint on the origin of HPAI H7N9 NA^3^,^6^. Using 7 newly sequenced HPAI H7N9 NA sequences to perform BLAST searches will always identify the NA sequences in A/mallard/Czech Republic/13438-29K/2010 (JF789604, H11N9) as the best hit, with an average identity of 96%. The A/wild bird/Korea/A3/2011 (JN244223, H7N9) NA only exhibits an average identify of 93% with HPAI H7N9 NAs. We observed that the alignment coverage of the Czech Republic sequence is 100%, while the Korea sequence can not be fully aligned with HPAI H7N9 NA (Fig. 6). Because mutation rates are usually not equal for transition (exchange of A <-> G or C <-> T) and transversion (C/T <-> A/G) in DNA sequences^27^, We also carefully analyzed the mutation numbers of the Czech Republic and Korea NA sequences. Totally, there are 34 and 42 transition with 2 and 35 transversion substitutions for the Czech Republic and Korea NAs, respectively. Thus, the sequence alignment suggested that the Czech Republic NA is more similar with the HPAI H7N9 NA. However, estimation of the evolutionary relationship based on the BLAST search is not reliable, because sequences can evolve faster or slower in different branches after divergence. Thus, the origins of HPAI H7N9 viruses should be determined by phylogenetic analysis, and our studies confirmed that Gao's results are more reliable for NA origin^6^.

For six internal genes, we didn't observe any LPAI H*x*N9 or H7N*x* (*x* is a number of 1–17 for HA and 1–10 for NA) genes from the phylogenetic trees (Supplementary Fig. S1e-S1j). Also, all of the six internal genes were found closely related to H9N2 viruses of poultries from Yangtze River delta. Thus, the reassortment of six internal genes is independent to HA and NA, and three potential models can be proposed with different reassortant orders of: 1) HA was first reassorted with NA then six internal genes; 2) NA was first reassorted with internal genes then HA; 3) HA was first reassorted with internal genes then NA. If the model 1 was correct, HA and NA were mostly reassorted in Zhejiang, since HAPI HA was derived from H7N3 in Zhenjiang duck. Because six internal HAPI genes were reassorted in the Yangtze river delta, the final reassortant step might also occur in this region, and the earliest patients would be diagnosed in more regions but not exclusively in Shanghai and Anhui. If the model 2 was correct, the reassortment of six internal genes and NA mostly occurred in the Yangtze river delta, while HA might be reassorted in Zhejiang. Thus, at least several earliest patients should be diagnosed in Zhejiang. However, the first patient in Zhejiang was infected on 25 March 2013, over one month later than the first infected patient in Shanghai (19 February 2013). More importantly, because the HPAI NA was not observed in poultries of Yangtze river delta, the reassortment of NA might be the last step. In this regard, two models are not reliable for interpreting the reassortment of HPAI H7N9 viruses. For the model 3, the reassortment of HA and six internal genes should most likely occur at least in the Yangtze river delta. In fact, wide-spreading H7N*x* and H9N2 viruses in this region provide opportunities for such reassortments.

**Figure 7:**
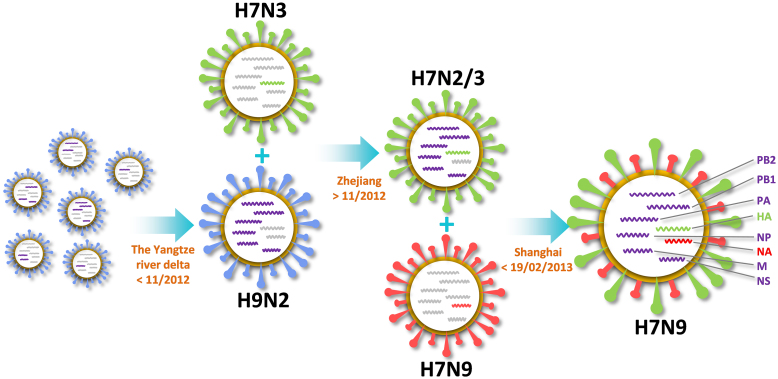
A “three-step reassortant” model of avian-origin H7N9 viruses. The six internal genes were reassorted from brambling and poultries in the Yangtze river delta before November 2012. HA of H7N3 was reassorted with the internal genes in poultries after November 2012. The final step for the reassortment of H7N9 NA from Korean wild bird occurred in Shanghai before 19/02/2013.

Based on currently available data and phylogenetic results, here we refined the Gao's “triple reassortant” model^6^ into a “three-step reassortant” model (Fig. 7): 1) The reassortment of six internal genes might occur in the Yangtze river delta, start before 11/2012 and still not finalize after the date, because A/brambling/Beijing/16/2012 was sequenced in November 2012 (From its annotation information); 2) HA of H7N3 subtype was reassorted with six genes of H9N2 subtype in Zhejiang Province after November 2012, because H7N9 subtype HA was not reassorted from A/brambling/Beijing/16/2012; 3) The reassortment of NA from H7N9 virus in Korean wild bird occurred in Shanghai before 19/02/2013, the date when the first Shanghai patient was infected. Our results can't fully exclude the probability of other models, if new data was reported. For example, Liu *et al*. argued that NS might have at least two origins based on newly released sequences^16^. However, this result made limited influence on our model, which compromised most of available evidences.

**Figure 8:**
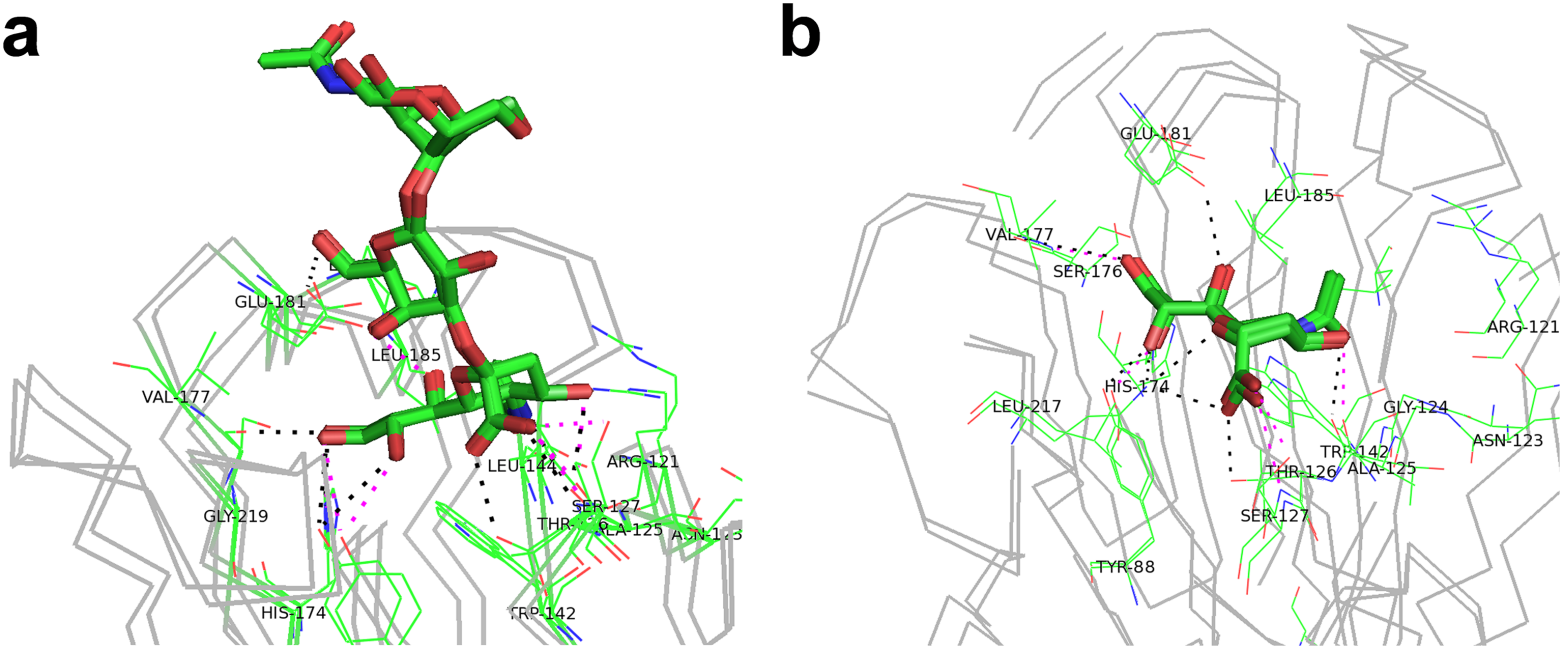
The modeled HA structures in complex with (a) avian receptor analog 3'SLN and (b) human receptor analog 6'SLN. The sticks represent the analog (3'SLN or 6'SLN) while the lines represent the HA. The complexes of NA-analog for A/Netherlands/219/2003 and A/Anhui/2013 were aligned together for comparison. The backbone of HA was shown in grey, while binding residues were labeled and their C atoms were highlighted in green. The hydrogen bonds were shown in black for A/Netherlands/219/2003 and magenta for A/Anhui/2013.

**Figure 9:**
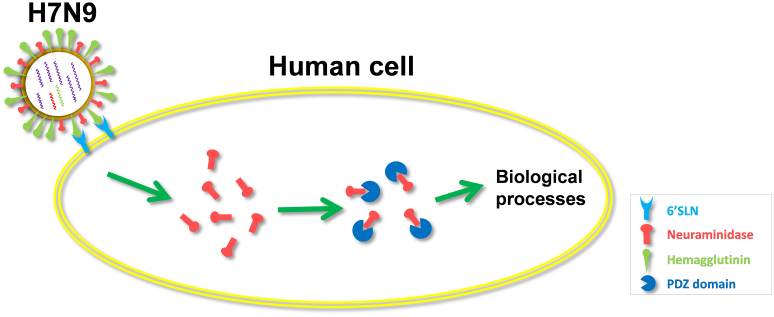
A model of HPAI H7N9 virus infection. The viruses enter human cells through the HA-6'SLN interaction, while both HA and NA further interact with PDZ domain proteins to influence biological processes.

Gao et al. and Kageyama et al. observed that genetic variations in HPAI H7N9 viruses have conferred the interaction between HA and avian-like receptors in humans^3^,^6^. Since the complex structures of H7N7 NA with avian and human receptor analogs were resolved separately^28^, we used the two structures as templates to model H7N9 NA in complex with two types of receptors (Fig. 8a,b). The results suggested that two modeled structures are highly similar with original complexes. In this regard, our results proposed a hypothetic model that how HPAI H7N9 viruses infect human cells (Fig. 9). First, HA binds to oligosaccharide 6'SLN of human receptors, mediates H7N9 virus uptake, and orchestrates membrane fusion. After viruses entered cells, NA will further interact with PDZ domain proteins through a C-terminal class 2 PDZ-binding motif to regulate the cellular processes (Fig. 9).

Taken together, beyond clarifying the controversial results of NA origin, our analyses further demonstrated that most variations in HPAI H7N9 viruses are not anti-viral resistant, whereas the current drugs are still effective for therapy. Moreover, the prediction and structural docking of epitopes will be useful for vaccine development. In addition, the high virulence of HPAI H7N9 viruses might be attributed to a newly evolved PDZ-binding motif at NA C-terminus. Our study is helpful for better understanding the virus and further experimental design.

## Methods

### Sequence data preparation

The nucleotide and protein sequences of 7 newly sequenced HPAI strains of H7N9 viruses were downloaded from the GISAID (Global Initiative on Sharing Avian Influenza Data) database on April 14. The 7 strains were isolated from 4 patients (A/Shanghai/1/2013 | EPI_ISL_138737, A/Shanghai/2/2013 | EPI_ISL_138738, A/Anhui/1/2013 | EPI_ISL_138739, and A/Hangzhou/1/2013 | EPI_ISL_138977), 2 birds (A/Chicken/Shanghai/S1053/2013 | EPI_ISL_138983, A/Pigeon/Shanghai/S1069/2013 | EPI_ISL_138985), and 1 environment (A/Environment/Shanghai/S1088/2013 | EPI_ISL_138984). Totally, we obtained 56 nucleotide sequences and 77 protein sequences for eight genes (HA, NA, PB2, PB1, PA, NP, M and NS) of H7N9 viruses. We also downloaded 253,527 nucleotide and 320,023 protein sequences of all influenza subtype viruses from the NCBI Influenza Virus Resource (ftp://ftp.ncbi.nih.gov/genomes/INFLUENZA/) on 9 April 2013^29^. To avoid any bias, only sequences annotated with “c” (complete sequence) were reserved. Moreover, CD-Hit^30^, a program of clustering similar sequences, was used to clear redundancy with a threshold of 100% sequence identity. Finally, the non-redundant NCBI dataset contains 115,192 nucleotide sequences.

### Phylogenetic analysis

Because too many influenza virus sequences are present and multiple alignments of all these sequences are quite time-consuming, here we adopted a simple approach for the phylogenetic analysis. For each gene, we used its nucleotide sequences of 7 newly sequenced strains to perform homologous searches in the non-redundant NCBI dataset, separately. Only top 200 hits were reserved for each sequence-based search. The results of 7 rounds of searches for each gene were merged together, while the redundant sequences were cleared. Totally, we obtained 208, 209, 215, 247, 220, 239, 209, and 214 non-redundant nucleotide sequences for HA, NA, PB2, PB1, PA, NP, M and NS, respectively. The Clustal Omega 1.1.0^31^ was chosen for multiple sequence alignments with default parameters for each gene, separately. The NJ trees were constructed with MEGA 5.10^32^. In addition, the MP trees were also constructed for HA and NA genes. The default parameters were used, while the number of Bootstrap Replications was selected as 1000.

To determine the TMRCA of the HPAI H7N9 outbreak, the molecular clock analysis was performed for each gene of HPAI H7N9 samples^11^. First, the nucleotide sequences of LPAI samples adjacent to HPAI virues in phylogenetic tree were picked out. Then, the maximum likelihood (ML) method in jmodeltest2^33^ was used for selecting an optimal substitution model for each gene, with default parameters. As previously described^11^, the temporal phylogenies were estimated with the parameters of exponential relaxed clock model, which allowed the different evaluation rates in different branches of each phylogenetic tree. Next, BEAST v1.7.5^34^, a tool for the analysis of molecular sequences based on the Bayesian MCMC approach, was used to analyze the TMRCA of the HPAI H7N9 outbreak. For each gene, we specified the chain length of 10 million steps in Bayesian MCMC sampling and removed a 10% “burn-in”^11^. Then, Tracer v1.5 (http://beast.bio.ed.ac.uk/Tracer) was applied to check and ensure promising distributions of parameters of posterior, prior probability and meanRate achieving moderate. Finally, FigTree v1.4.0 (http://beast.bio.ed.ac.uk/FigTree) was used to visualize the phylogenetic trees and mark the TMRCA of each gene.

### Non-synonymous variation analysis

We first analyzed non-synonymous variations between patient and non-pathogenic samples. Because the results of multiple sequence alignments exhibited that the sequences of A/Shanghai/1/2013 are quite different from other patient samples, we separated 4 patient samples into two groups, including the A/Shanghai/1/2013 group and the second group. The sequences of A/Anhui/1/2013 were selected as the representative sequences for the second group. Based on phylogenetic results, the nearest non-pathogenic samples were chosen as benchmark sequences for comparison. Again, because the NA sequences among Korean wild birds are highly divergent, we further aligned 5 NA sequences from Korean wild bird H7N9 viruses (A/wild bird/Korea/A14/11 | EPI_ISL_120868, A/wild bird/Korea/A3/11 | EPI_ISL_120869, A/spot-billed duck/Korea/447/11 | EPI_ISL_120871, A/wild bird/Korea/A9/11 | EPI_ISL_120881, and A/wild duck/Korea/SH20-27/2008 | EPI_ISL_133001). The identical positions in the alignment results were adopted as a benchmark for identifying NA variations in patient samples.

### Structural modeling

Modeller 9.11^35^,^36^ was employed for homology modeling the structures of HA, NA and the complexes. From the PDB database^37^, previous reported H7 hemagglutinin structures including 4DJ6, 4DJ7, 4DJ8 from A/Netherlands/219/2003 (H7N7)^28^ were downloaded and used as the templates for modeling HA monomer, HA in complex with avian receptor analog (3'-sialyl-N-acetyllactosamine [3'SLN]), and HA in complex with human receptor analog (6'-sialyl-N-acetyllactosamine [6'SLN]) in H7N9 virus. To dissect the key residues of NA for interacting drugs including Oseltamivir, Zanamivir and Laninamivir, known complex structures of 2QWK^17^, 3TI3^18^ and 3TI5^18^ were selected as the templates for modeling NA-Oseltamivir, NA-Laninamivir and NA-Zanamivir complexes in H7N9 virus, respectively. The HA and NA protein sequences of A/Anhui/1/2013 were chosen as the representative sequences for structural modeling. All the modeled structures were validated by Procheck^38^ and visualized in PyMOL (http://www.pymol.org/), an open-source molecular visualization tool. With default parameters in PyMOL, the atoms were shown in different colors (C: green; N: blue; O: red).

### Epitope prediction and docking

The CTL epitope predictor NetCTL 1.2 Server^22^ was used for the prediction of MHC class I binding peptides for all HA protein sequences of 7 newly sequenced strains of H7N9 viruses. The B44 supertype was chosen, while the default parameters were adopted. Because the sequence-based prediction is not reliable and false positive hits can not be avoided, we additional performed structural modeling and docking analyses. Since the predicted epitopes are identical across the 7 strains, here the HA protein sequence of A/Anhui/1/2013 was chosen for further analyses. The 3D structures of HLA-B*4405 (3DX8) and DM1-TCR in complex with HLA-B*4405 (3DXA) were downloaded from the PDB database^37^. As previously described^19^, we used ClusPro 2.0^39^ to dock all predicted epitopes to HLA-B*4405, and then regarded the HLA-B*4405/epitope complex as an integrated ligand and further docked it to DM1-TCR. The binding energy scores were automatically calculated by ClusPro^39^.

## Author Contributions

Y.X. designed and supervised experiments. Y.W. performed phylogenetic analysis. T.G., X.L., Y.Y. and J.R. repeated and validated phylogenetic results. H.C. and W.D. performed variation analysis. Z.L. performed structural modeling of HA and NA. Z.P. performed epitope prediction and docking. Z.D. performed PDZ-binding analysis. Y.X. and Z.L. wrote manuscript with contributions of all authors. All authors reviewed the manuscript.

## Supplementary Material

Supplementary Information Supplementary Figure 1

## Figures and Tables

**Table 1:** The TMRCA of each gene in HPAI H7N9 viruses. The values with 95% credible intervals were present in parentheses

Gene	TMRCA of HPAI H7N9 viruses	Duration of unsampled diversity (years)	Mean evolutionary rate *10^−3^ (subst. per site per year)
HA	Oct 2011 (Jun 2010, Sep 2012)	3.27 (1.55, 4.89)	4.81 (2.20, 7.95)
NA	Sep 2011 (Mar 2010, Sep 2012)	2.78 (1.14, 4.21)	5.21 (3.56, 7.01)
M	Jan 2012 (Mar 2011, Sep 2012)	1.38 (0.69, 2.69)	4.68 (2.58, 6.73)
NP	May 2011 (May 2010, Jul 2012)	0.67 (0.00, 2.47)	4.93 (2.95, 6.96)
NS	May 2012 (Aug 2011, Nov 2012)	1.48 (0.72, 2.18)	4.44 (2.56, 6.40)
PA	Jul 2012 (Dec 2011, Nov 2012)	0.89 (0.24, 1.48)	7.77 (3.87, 11.48)
PB1	Jan 2012 (Feb 2011, Sep 2012)	1.19 (0.23, 2.44)	4.06 (2.42, 5.69)
PB2	Mar 2012 (Sep 2011, Sep 2012)	0.63 (0.12, 1.20)	5.15 (3.06, 7.26)

**Table 2:** The key residues of NA-drug interaction. The numbers indicate the distances which are given in Å. The distances larger than 4 Å were not shown for residues

Residue	Oseltamivir^a^	Oseltamivir^b^	Zanamivir	Laninamivir	Variation^c^
R118	2.8	3.4	3.4	3.0	No
E119	2.8	3.3	3.2	2.8	No
D151	2.6	2.9	3.0	3.0	No
R152	2.6	2.9		3.0	No
R156			3.6		No
W178	3.7	3.9	3.2	2.7	No
I222	3.9	3.8	3.7	3.7	No
R224	3.9	3.4	4.0		No
E227			3.2	3.1	No
A246			3.4	3.2	No
E276	3.5	3.4	2.9	2.9	No
E277	3.7		4.0	3.8	No
R292	3.2	3.3	3.3	2.9	R- > K^d^
N294	3.8		3.2	3.3	No
R371	2.7	3.4	3.1	3.2	No
Y406	3.4	3.2	3.1	3.2	No

*^a^*From the original NA-Oseltamivir complex (2QWK).

*^b^*From the H7N9 NA-Oseltamivir model.

*^c^*The variation at interacting residues.

*^d^*The R292K variation in A/Shanghai/1/2013 patient sample.

**Table 3:** The predicted epitopes of HA and their docking results to HLA-B*440 and DM1-TCR. The predicted score of NetCTL 1.2^22^ was shown for each epitope. The binding energy scores were given by ClusPro^39^. A lower energy value means a more stable docking complex^19^

			To HLA-B*4405	To DM1-TCR
Start	Epitope	NetCTL	Energy	Energy
88	LEFSADLII	1.7827	−904.6	−643.9
353	WEGLIDGWY	0.8101	−821.1	−804.3
333	PEIPKGRGL	1.0328	−683	−703.4
502	REEAMQNRI	1.4055	−637.4	−757.9
412	VEKQIGNVI	1.0442	−609	−756.3
112	NEEALRQIL	1.525	−508.5	−756.4
441	MENQHTIDL	1.9779	−534.5	−752.3
469	AEEDGTGCF	1.3287	−454.1	−652.3

**Table 4:** The statistics of PDZ-binding motifs in H5N1 and H7N9 virus proteins. Although 52.9% of H7N9 NS1 genes contain a class 2 binding motif, this motif is not present in any HPAI H7N9 viruses. The class 3 motif was not present in any H7N9 proteins

	H5N1								H7N9					
	Class 1		Class 2		Class 3		Total		Class 1		Class 2		Total	
Protein	*Num.*	*Per.*	*Num.*	*Per.*	*Num.*	*Per.*	*Num.*	*Per.*	*Num.*	*Per.*	*Num.*	*Per.*	*Num.*	*Per.*
NA	0	0	0	0	0	0	1352	0	0	0	20	95.2%	21	95.2%
NS1	901	87.9%	0	0	1	0.1%	1025	88.0%	9	52.9%	0	0	17	52.9%
HA	0	0	1598	98.3%	1	0.1%	1625	98.4%	0	0	22	100%	22	100%
PB1	0	0	0	0	0	0	831	0	0	0	0	0	15	0
PB2	0	0	0	0	0	0	965	0	0	0	0	0	19	0
PA	0	0	0	0	0	0	1019	0	0	0	0	0	20	0
M1	0	0	0	0	0	0	312	0	0	0	0	0	4	0
M2	0	0	0	0	0	0	382	0	0	0	0	0	9	0
NP	0	0	1	0.2%	1	0.2%	595	0.3%	0	0	0	0	20	0
NS2	0	0	2	0.5%	0	0	416	0.5%	0	0	0	0	12	0
PB1-F2	0	0	0	0	0	0	320	0	0	0	0	0	13	0
