# Supplementary material for: Towards a better understanding of the novel avian-origin H7N9 influenza A virus in China
Source: Sci Rep. 2013 Jul 30;3:2318. doi: 10.1038/srep02318 (PMC3727058; doi:10.1038/srep02318)

Towards a better understanding of the novel avian-origin H7N9 influenza A virus in China

Yongbo Wang[1,†], Zhangyan Dai[2,†], Han Cheng[1,†], Zexian Liu[1,†], Zhicheng Pan[1,†], Wankun Deng[1,†], Tianshun Gao[1,†], Xiaotong Li[1], Yuangen Yao[1], Jian Ren[3], Yu Xue[1,*]

[1]Department of Biomedical Engineering, College of Life Science and Technology, Huazhong University of Science and Technology, Wuhan, Hubei 430074, China

[2]School of Chemistry and Molecular Biosciences, University of Queensland, Brisbane, Queensland 4027, Australia

[3]State Key Laboratory of Biocontrol, School of Life Sciences, Sun Yat-sen University, Guangzhou, Guangdong 510275, China

[†]These authors contributed equally to this work.

*Correspondence and requests for materials should be addressed to Yu Xue, Tel: +86-27-87793903, Fax: +86-27-87793172, E-mail: xueyu@hust.edu.cn.

Supplementary information

Supplementary Fig. S1 – Phylogenetic analyses of H7N9 virus genes. The detailed MP trees for (a) HA and (b) were presented, while the NJ trees were also constructed for (c) HA and (d) NA. The NJ trees were also shown for (e) PB1, (f) PB2, (g) PA, (h) NP, (i) M and (j) NS. The HPAI viruses in patients were marked in red, while HPAI viruses from non-human samples were marked in blue. The nearest genes from LPAI viruses were shown in green.

Supplementary Figure 1a

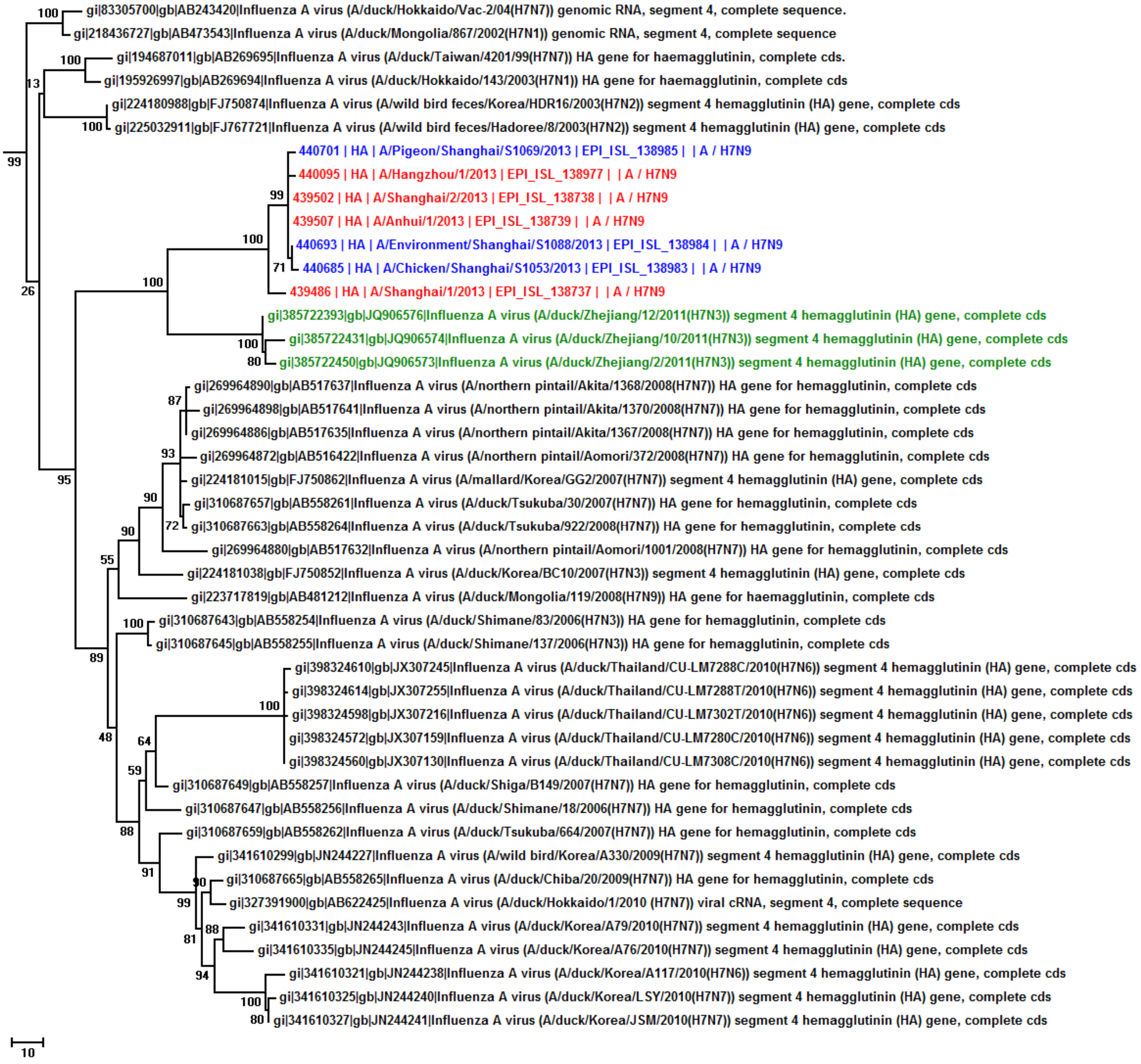

Supplementary Figure 1b

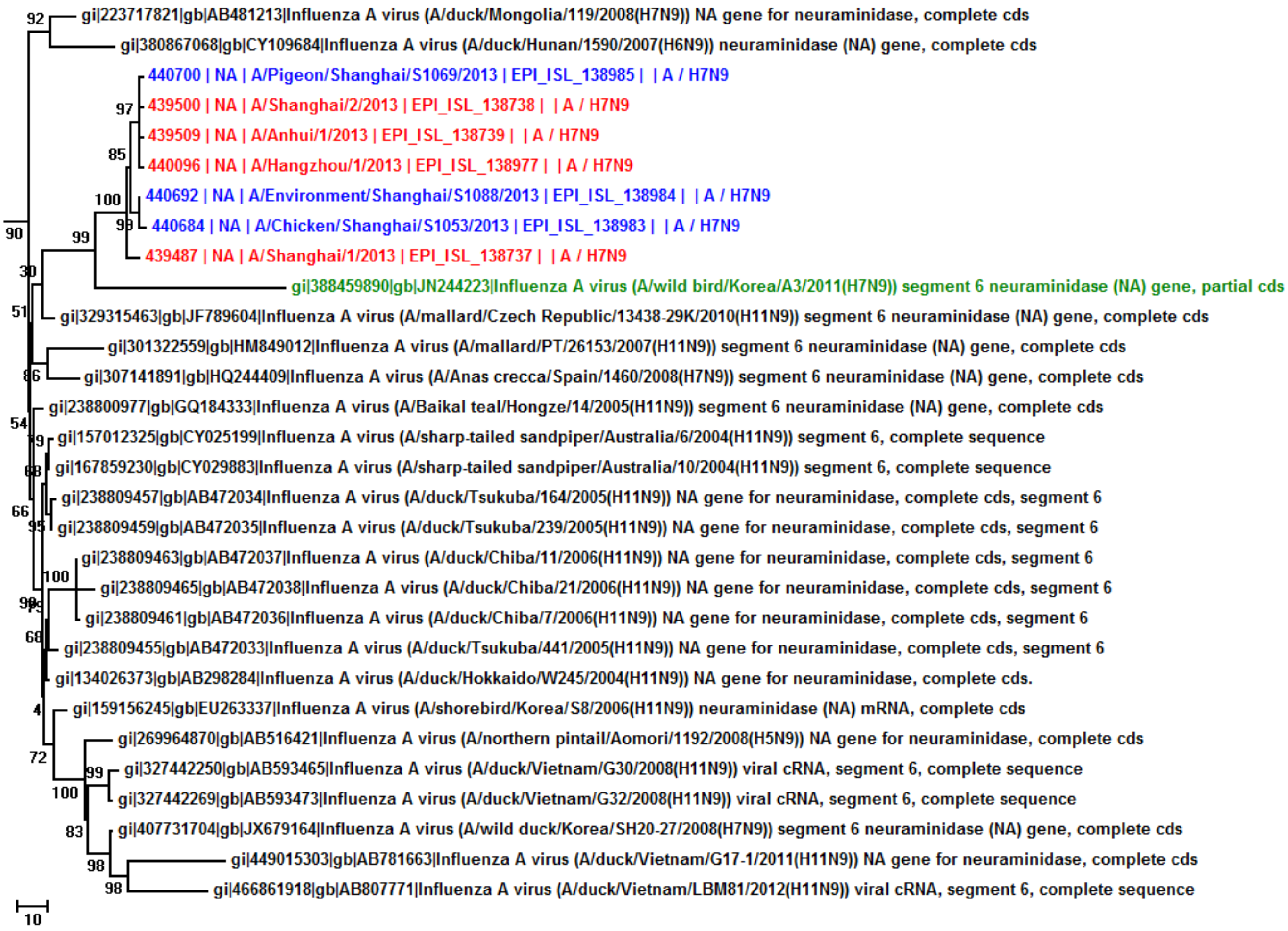

Supplementary Figure 1c

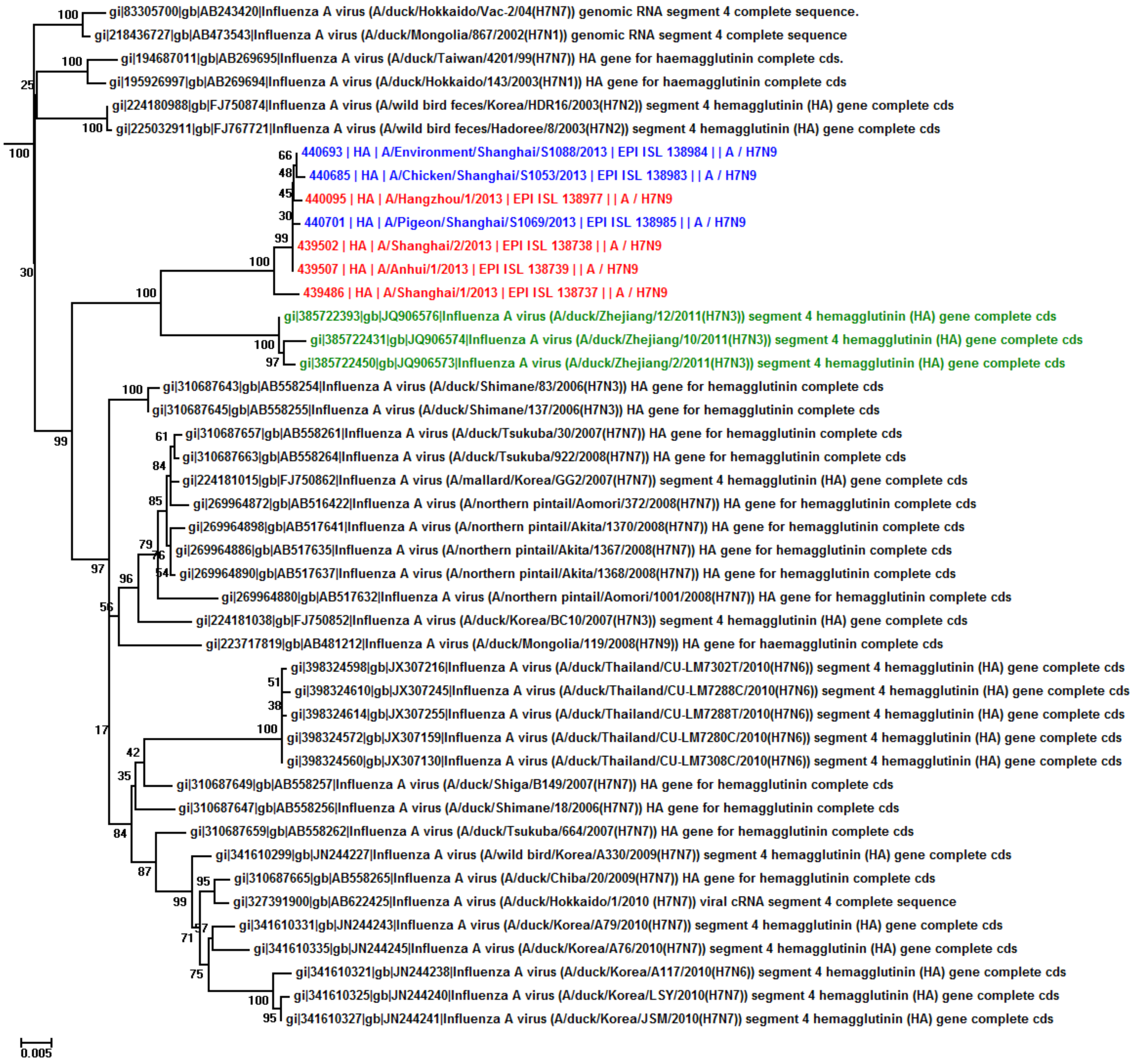

Supplementary Figure 1d

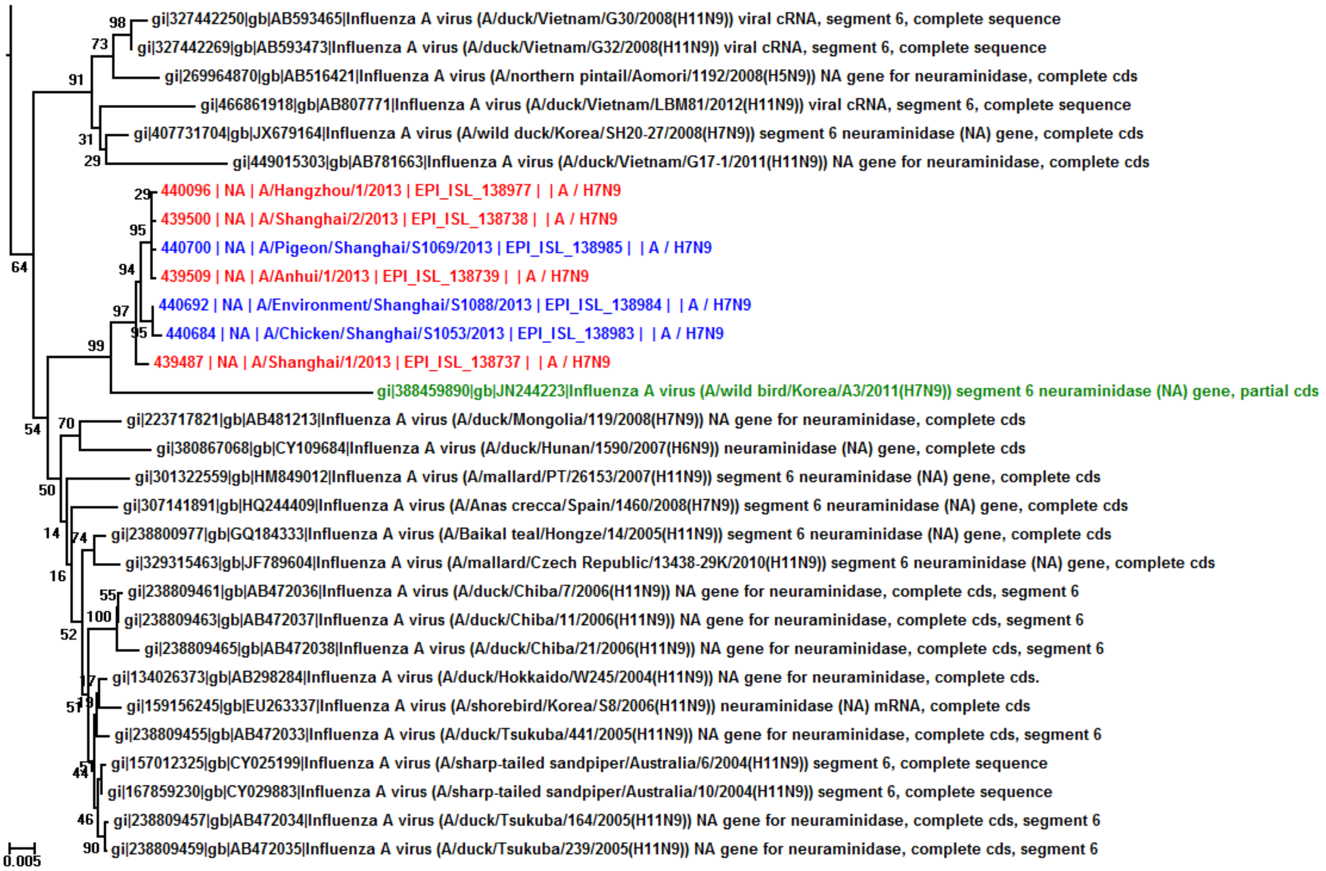

Supplementary Figure 1e

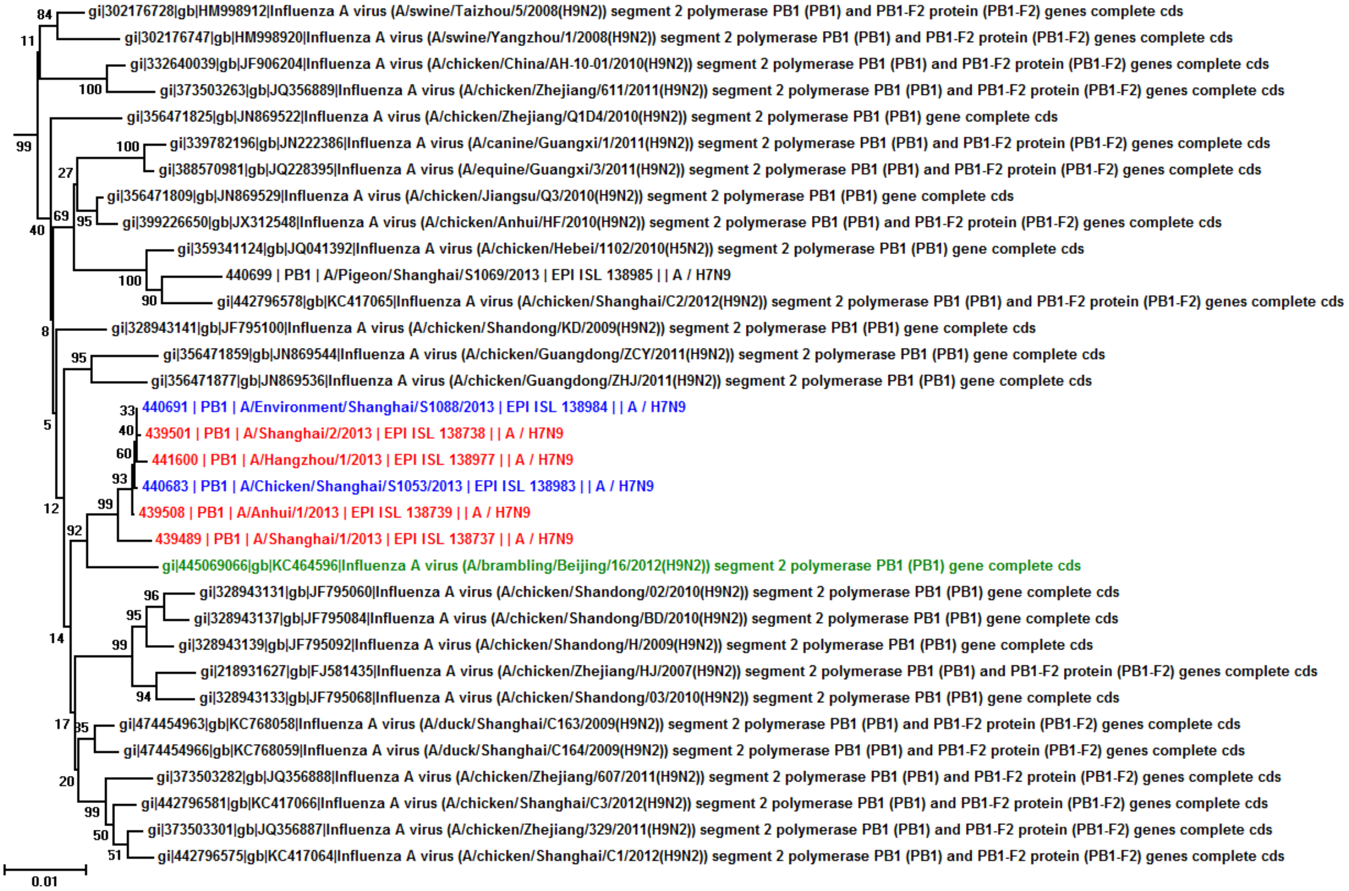

Supplementary Figure 1f

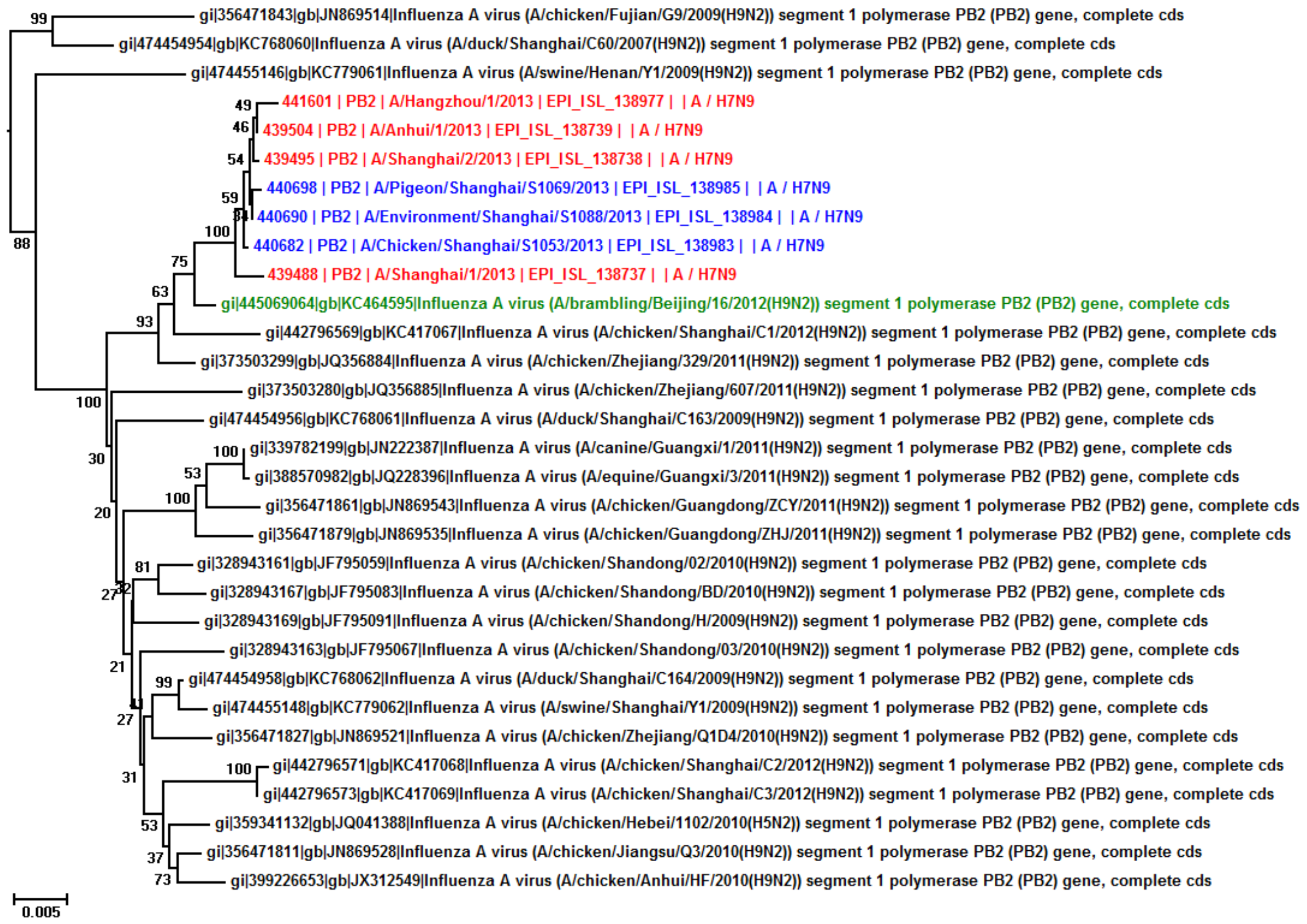

Supplementary Figure 1g

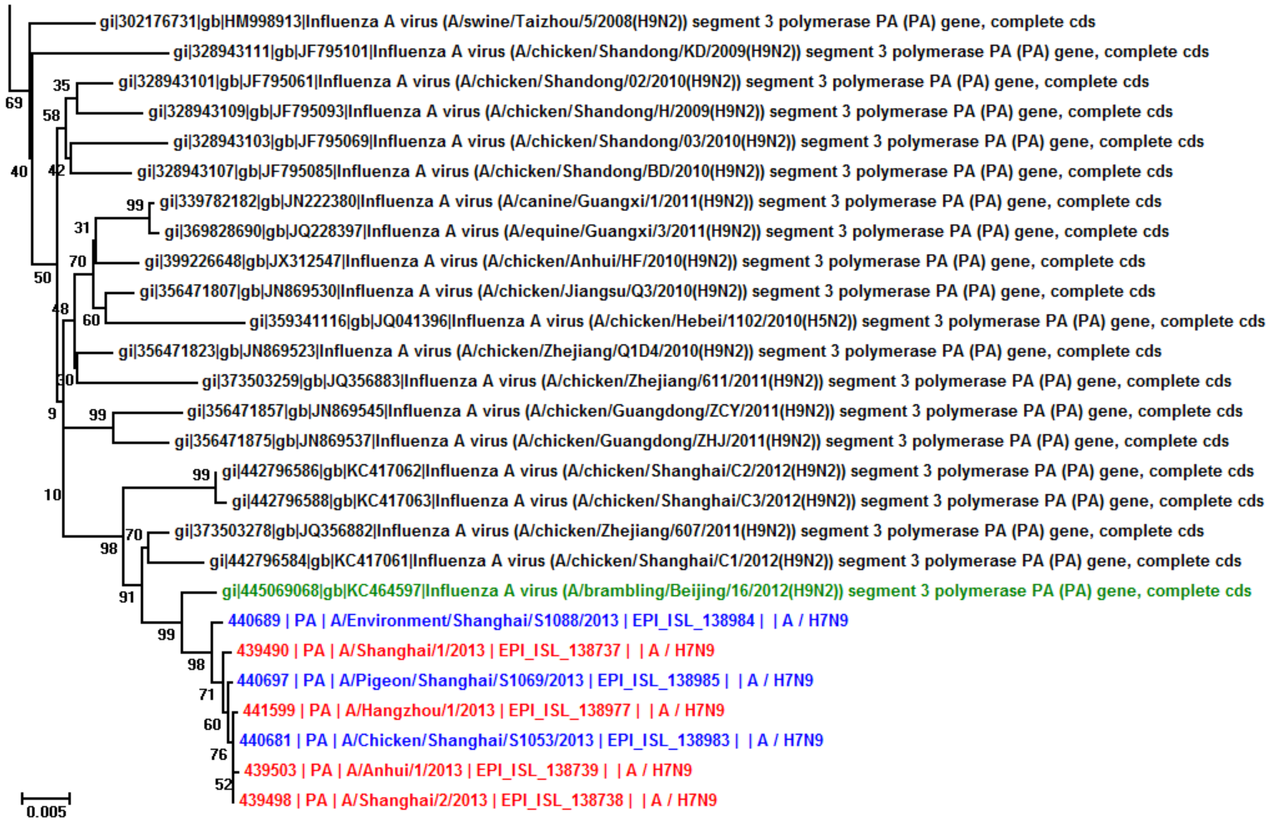

Supplementary Figure 1h

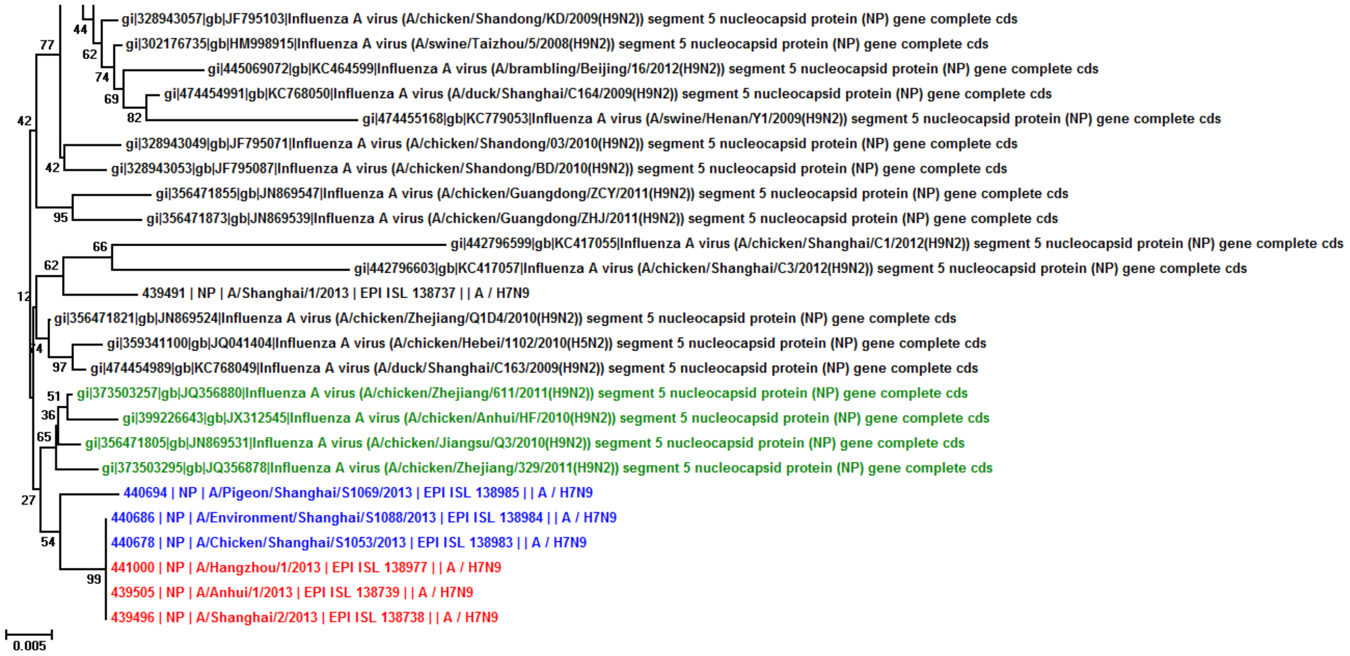

Supplementary Figure 1i

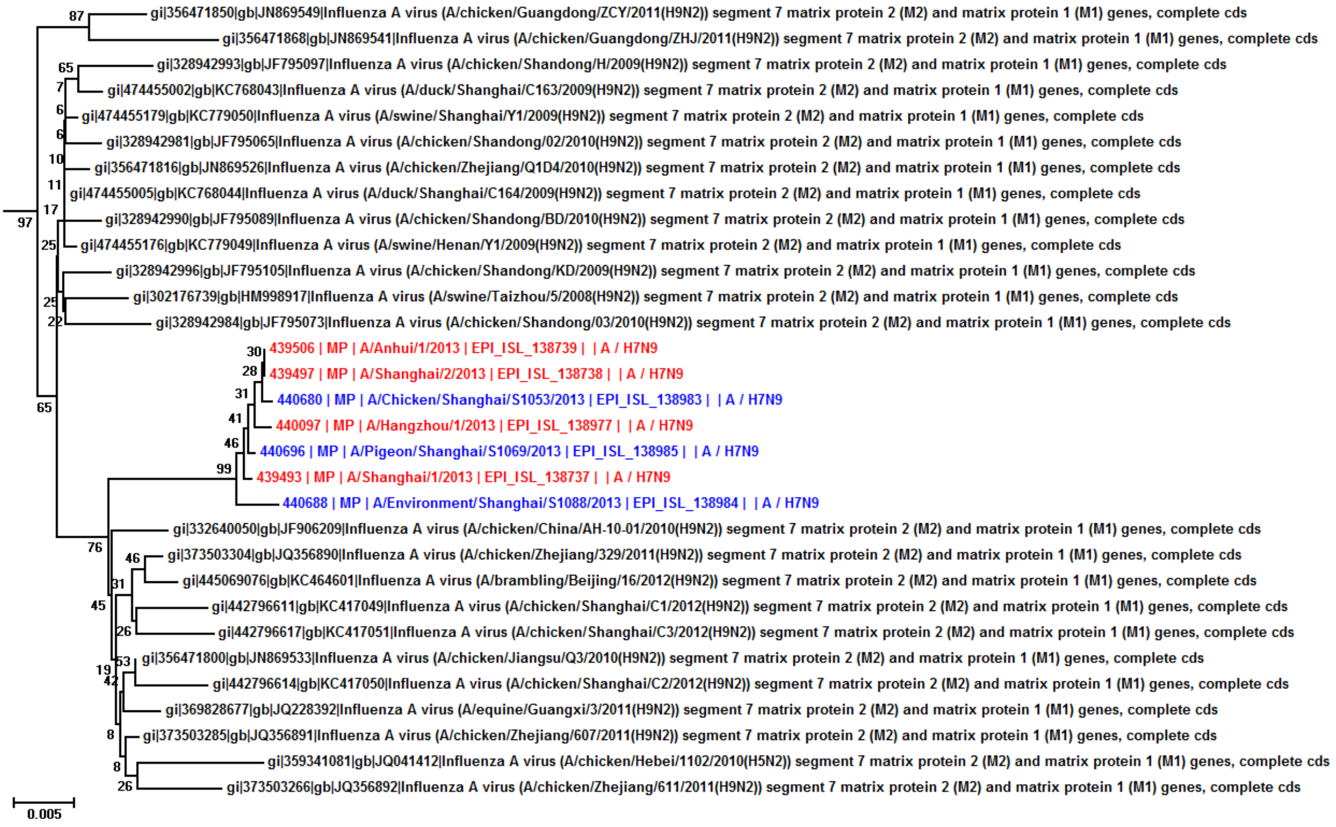

Supplementary Figure 1j

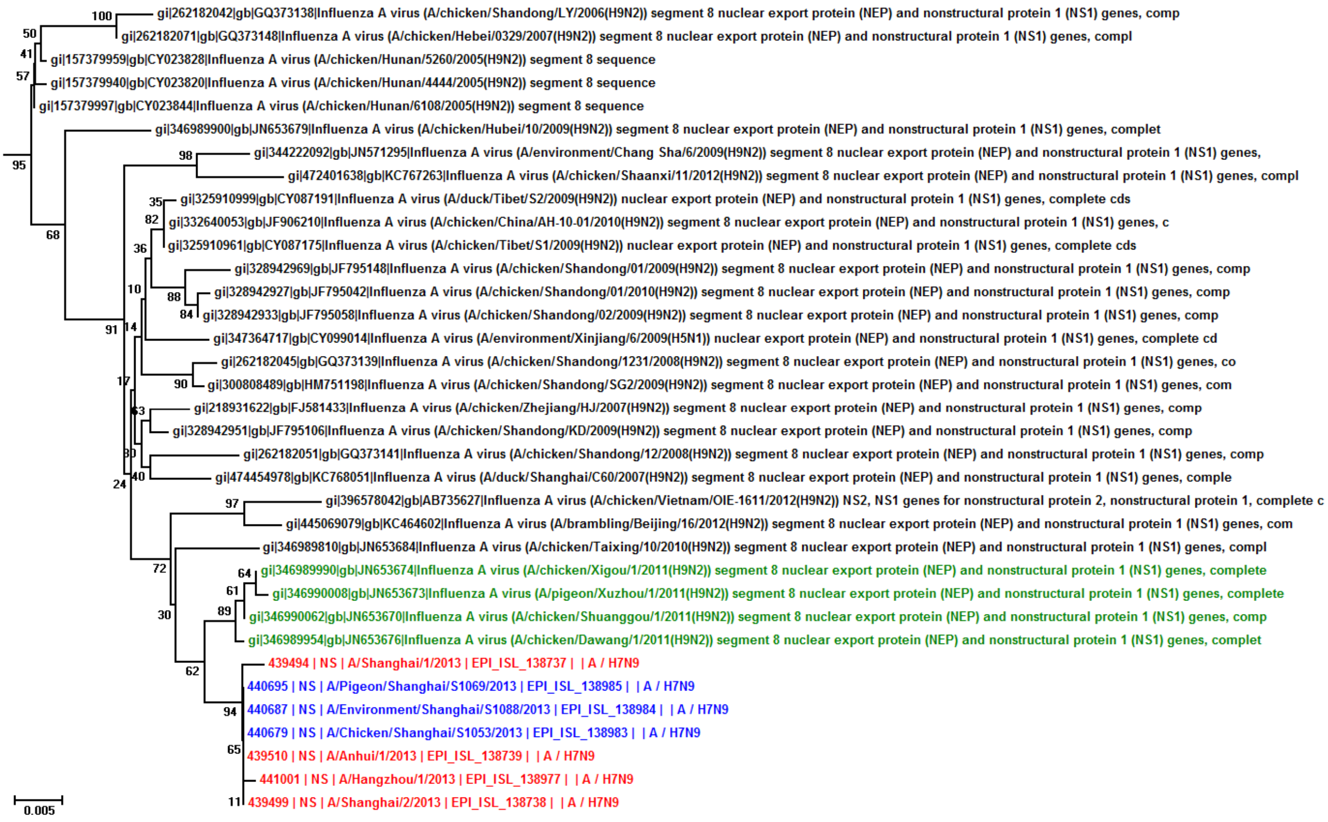

Supplement: Supplementary Information — Supplementary Figure 1 [file srep02318-s1.pdf]
